# Synthesis, Clastogenic and Cytotoxic Potential, and In Vivo Antitumor Activity of a Novel N-Mustard Based on Indole-3-carboxylic Acid Derivative

**DOI:** 10.3390/molecules30183710

**Published:** 2025-09-12

**Authors:** Marina Filimonova, Olga Soldatova, Anna Shitova, Valentina Surinova, Vitaly Rybachuk, Alexander Kosachenko, Kirill Nikolaev, Daria Filatova, Ekaterina Prosovskaya, Sergey Ivanov, Petr Shegay, Andrey Kaprin, Alexander Filimonov

**Affiliations:** 1 A. Tsyb Medical Radiological Research Center—Branch of the National Medical Research Radiological Center of the Ministry of Health of the Russian Federation, 249036 Obninsk, Russia; ovsoldatova97@gmail.com (O.S.); annaredrose@mail.ru (A.S.); val_surinova@mail.ru (V.S.); br.shepard@list.ru (A.K.); nireallki@gmail.com (K.N.); filatovadaria.nik@gmail.com (D.F.); ekaterina.prosovskaya@gmail.com (E.P.); oncourolog@gmail.com (S.I.); filimonov_alex@mail.ru (A.F.); 2 National Medical Research Radiological Center of the Ministry of Health of the Russian Federation, 249036 Obninsk, Russia; dr.shegai@mail.ru (P.S.); kaprin@mail.ru (A.K.); 3 Medical Institute (RUDN University), Peoples’ Friendship University of Russia, 117198 Moscow, Russia

**Keywords:** nitrogen mustard, indole-3-carboxylic acid derivative, alkylating agent, TLR agonist, clastogenic effect, cytotoxic effect, antitumor effect, conventional chemotherapy, metronomic chemotherapy

## Abstract

Compound T1089—a novel nitrogen mustard based on an indole-3-carboxylic acid derivative (ICAD)—has been synthesized. The ICAD used as the basis for T1089 is a TLR agonist capable of activating an antitumor immune response. This study describes the synthesis method and presents the results of preliminary investigations of this compound. This research included an assessment of acute toxicity in mice, in vivo clastogenic activity evaluated via the bone marrow chromosome aberration (BMCA) test in mice, in vitro cytotoxicity determined by the MTT assay against human lung carcinoma A549 cells, and in vivo antitumor effects (ATEs) in models of conventional chemotherapy (CCT) of solid tumors in mice. The bifunctional alkylating agent cyclophosphamide (CPA) was used as a reference drug. **Toxicological** studies revealed that T1089 belongs to toxicity class III (moderately toxic), with acute toxicity values (LD_16_ and LD_50_) in mice following intraperitoneal (i.p.) administration being 191 and 202 mg/kg, respectively. The alkylating activity and clastogenic potential of T1089 were demonstrated by its effects in the BMCA test, which were comparable to those of CPA. A single i.p. administration of CPA and T1089 at a dose of 0.064 mmol/kg induced similar stimulation of structural mutagenesis associated with DNA strand breaks. The frequency of karyocytes with aberrations increased 20-fold compared to the control, primarily due to a rise in chromatid breaks and fragments, and to a lesser extent, due to an increase in exchange-type aberrations. **In vitro** cytotoxicity studies indicated differences in the mechanisms of alkylating activity between CPA and T1089. According to the MTT assay, the cytotoxic effects of CPA were observed only at concentrations exceeding 2 mM (IC_50_ = 4.2 ± 0.3 mM), corresponding to lethal in vivo doses, which is expected since the formation of CPA’s alkylating metabolite requires hepatic microsomal enzymes. In contrast, significant cytotoxic effects of T1089 were observed at much lower concentrations (15–50 μM, IC_50_ = 33.4 ± 1.3 μM), corresponding to safe in vivo doses. **Differences** were also observed in the in vivo ATEs of CPA and T1089 in the Ehrlich solid carcinoma (ESC) CCT model. Following seven i.p. administrations at 48 h intervals (33 mg/kg), both compounds exhibited increasing toxicity, manifested as cumulative body weight loss in treated mice. However, despite the aggressive CCT regimen, ESC showed low sensitivity to CPA. The ATE of CPA developed slowly, reaching a significant level only after four injections, and even after seven administrations, tumor inhibition (TI) did not exceed 30%. In contrast, ESC was significantly more sensitive to T1089 under the same CCT conditions. The ATE of T1089 exhibited a cumulative pattern but developed more rapidly and to a greater extent. A significant antitumor effect was observed after just two injections, with maximal efficacy (TI = 53%) achieved after four injections and sustained until the end of the observation period. **A high** ATE of T1089 was also observed in the B-16 melanoma CCT model. Following six i.p. administrations at 48 h intervals (28 mg/kg), T1089 treatment was associated with minimal toxicity. Despite this mild CCT regimen, melanoma exhibited high sensitivity to T1089. Maximal ATE (TI = 56%) was achieved after two injections, and subsequent administrations maintained a consistently high efficacy (TI = 52–55%) until the end of the study. **In summary**, preliminary findings demonstrate that T1089 possesses alkylating activity characteristic of bifunctional agents, accompanied by high in vitro cytotoxicity and in vivo ATEs in CCT models (at high doses). Given that the ICAD used as the basis for T1089 is a TLR agonist capable of stimulating antitumor immunity, T1089 can be considered a dual-action alkylating agent with combined antitumor effects. These results justify further investigation of T1089 in conventional and metronomic chemotherapy regimens, particularly in combination with immune checkpoint inhibitors and antitumor vaccines.

## 1. Introduction

Cancer remains one of the most severe social and medical challenges, posing a significant threat to global health. According to the International Agency for Research on Cancer (IARC), approximately 20 million new cancer cases and 9.7 million cancer-related deaths were reported worldwide in 2022. By 2050, the global cancer burden is projected to rise to 35 million cases [1]. To counter these alarming trends, it is essential to strengthen preventive measures against cancer risk factors, advance innovative approaches such as targeted therapy, immunotherapy, epigenetic therapy, and genomic and molecular profiling, as well as further refine conventional cancer treatments, including surgery, radiotherapy, and chemotherapy [2,3,4,5,6,7].

Chemotherapy for cancer has a long history spanning over 80 years. The oldest class of anticancer agents is alkylating agents (AAs). Their development began with the discovery of the antitumor activity of nitrogen mustard. Initially, AAs were used as chemical weapons during World War I, causing severe vesicant effects accompanied by bone marrow aplasia and pancytopenia [8,9]. These observations led to trials of mustard gas as an anticancer agent. By 1931, sustained positive responses were demonstrated in mouse tumor models and in patients with skin cancer and sarcomas [10]. A second impetus came in December 1943 when sulfur mustard was accidentally released in the Italian harbor of Bari. The victims exhibited severe lymphoid hypoplasia and myelosuppression, prompting further research into the antitumor effects of nitrogen mustards. Encouraging clinical results in leukemia and lymphoma treatment, published in 1946–1947 [11,12,13,14], became a cornerstone in cancer chemotherapy development. In March 1949, the USFDA approved the first alkylating drug, mechlorethamine. Subsequent efforts focused on developing new nitrogen mustard derivatives and other classes of alkylating compounds. Seventy-five years later, AAs remain among the most widely used pharmacological agents in chemotherapy for many malignancies [15,16].

AAs are electrophilic compounds that react with nucleophilic DNA sites (ring N and extracyclic O atoms in DNA bases), leading to the covalent transfer of an alkyl group [15,17]. Covalent adducts can vary from simple methyl groups to complex alkyl additions. DNA damage—determined by the number of reactive sites in AAs (monofunctional or bifunctional agents), the type of nucleophilic substitution (SN1 or SN2 type), the nature of the transferred alkyl group (methyl, chloroethyl, etc.), and the DNA structure (double- or single-stranded)—can result in abnormal base pairing, intrastrand or interstrand crosslinks, and single- or double-strand breaks [17,18,19]. Such damage disrupts critical DNA functions, including replication and transcription, defining the mutagenic and cytotoxic potential of AAs.

Under such mechanisms of alteration leading to the cytotoxic action of alkylating agents (AAs), as well as to the effects of ionizing radiation, which also damages DNA, the most sensitive tissues are those that are actively proliferating. This enables the achievement of therapeutic effects in chemotherapy with AAs for actively growing malignant neoplasms. However, it also creates conditions for the development of systemic genotoxic, mutagenic, and carcinogenic effects, as well as hematological or non-hematological organ-specific complications [20,21,22,23,24,25,26].

In this regard, from their discovery to the present day, AAs have remained the subject of intensive research efforts aimed at developing safer and more effective agents. Currently, various approaches are employed to improve the therapeutic profile of AAs, including structural modifications of synthetic and natural AAs, as well as the development of new alkylating therapy technologies. To enhance targeted delivery, tumor selectivity, and cytotoxic efficacy, research is being conducted on hypoxia-activated and tumor-associated enzyme-activated bioreductive alkylating prodrugs, conjugates of AAs with antibodies and ligands for tumor receptors, conjugates of AAs with tumor-targeting pharmacophores, steroid-based AAs for hormone-sensitive tumors, DNA-targeted AAs specific to the minor groove, novel technologies for antibody-directed and gene-directed enzyme alkylating therapy, and synergistic modulation of DNA alkylation-induced signaling pathways [15,16,19,27,28,29,30].

Moreover, in recent decades, there has been a significant revision of alkylating therapy strategies themselves [31,32,33]. The traditional strategy, aimed at maximizing cytotoxic effects on tumor cells, typically involves administering AAs at doses close to the maximum tolerated dose (MTD). This approach is associated with a high toxicity profile, necessitating intermittent chemotherapy with sufficiently long treatment-free intervals to allow patients to recover from acute toxic effects. However, such breaks between chemotherapy cycles also create conditions for tumor recovery and the development of resistance, limiting treatment efficacy [34,35].

As early as the 1970s, data emerged indicating that immune factors significantly contribute to the effects of AAs, leading to the formulation of the concept of their dual antitumor action: direct cytotoxic effects and indirect effects mediated by the activation of antitumor immunity [36,37]. Furthermore, it was established that immunomodulatory doses of AAs are generally significantly lower than their cytotoxic doses [38]. By the early 2000s, the strategy of metronomic chemotherapy was proposed, involving frequent (sometimes daily) administration of AAs at doses well below the MTD, without prolonged treatment interruptions [39].

In metronomic therapy with AAs, antitumor effects arise from their impact on various cells in the tumor microenvironment and are mediated by anti-angiogenic action (reduction in circulating endothelial progenitor cells, stimulation of TSP1 secretion, and apoptosis of proliferating endothelial cells) and the induction of T-cell-dependent immune responses (inhibition of immunosuppressive Treg cells, decreased levels of immunosuppressive cytokines, recovery of IFN-γ-producing NK cells, polarization of Th1 and Th17 lymphocytes and macrophages, and activation of dendritic cells) [40,41,42,43,44,45,46]. Currently, AAs-based metronomic chemotherapy is being clinically investigated for various solid tumors and hematological malignancies, either alone or in combination with other anticancer agents (anti-angiogenic drugs, immune modulators, immune checkpoint inhibitors, vaccines, radiotherapy, and conventional anticancer drugs) [31,33,47]. Many of these studies show promising results, suggesting that harnessing the immune system to confer dual antitumor action to chemotherapy is a viable therapeutic strategy.

The Laboratory of Radiation Pharmacology at the A.F. Tsyb Medical Radiological Research Center has long been engaged in the chemistry and pharmacology of indole-3-carboxylic acid derivatives. In recent years, we have synthesized a series of new aminoalkyl esters of 5-methoxyindole-3-carboxylic acid exhibiting antiviral and interferon-inducing activity [48,49,50]. In a macrophage-like cell model, it was demonstrated that these indole-3-carboxylic acid derivatives (ICADs) activate genes of Toll-like receptors (TLRs) in innate immunity [51]. Moderate stimulation of TLR2, TLR7, TLR8, and TLR9 genes was observed, along with a substantial (6.5- to 16,000-fold) increase in the expression of TLR3 and TLR4 genes, accompanied by elevated expression of interferon genes (IFNA1, IFNA2, IFNB1, IFNK, and IFNL1), interferon receptor genes (IFNAR1 and IFNAR2), and signaling molecule genes (JAK1 and ISG15), which mediate interferon effects. Additionally, there was a several-fold increase in the expression of cytokine genes (TNFA, IL6, IL12A, and IL12B). These findings indicated that the studied ICADs activated TLR genes of innate immunity, triggered pathogen recognition signaling mechanisms, and stimulated the synthesis of interferons and pro-inflammatory cytokines, leading to an antiviral state in cells. This conferred a broad spectrum of antiviral activity against influenza viruses (H1N1, H2N2, and H3N2), herpes simplex virus type 1, encephalomyocarditis virus, and SARS-CoV-2 coronavirus [48,49,50].

At the same time, recent data suggest that TLR agonists, by inducing acute inflammatory responses, stimulate dendritic cell maturation and antigen presentation, thereby activating antitumor immune responses and creating prospects for their use in combination with immune checkpoint inhibitors and antitumor vaccines [52,53,54]. The antitumor activity of our synthesized ICADs has been preliminarily investigated [55]. In in vivo tumor models, repeated intraperitoneal administration (at 72 h intervals) of the tested ICADs at doses of 1/4 LD_16_ resulted in moderate (20–40%) inhibition of solid Ehrlich carcinoma and mouse cervical cancer growth and significant (50–60%) suppression of Lewis lung carcinoma metastasis. Currently, we are planning detailed studies on the effects of these compounds on tumor cells, the tumor microenvironment, and immune and inflammatory responses in neoplastic tissues.

**Figure 1:**
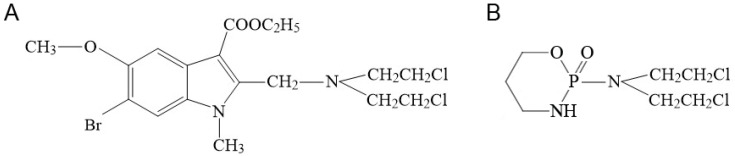
Structural formulas of the studied compounds. (**A**) Compound T1089–1-methyl-2-[bis(2-chloroethyl)aminomethyl]-3-carbethoxy-5-methoxy-6-bromoindole. (**B**) Cyclophosphamide (CPA)–2-[bis(2-chloroethyl)amine]tetrahydro-2*H*-1,3,2-oxazaphosphorine-2-oxide.

Concurrently, having confirmed that the immunomodulatory activity of the studied ICADs can inhibit tumor growth and metastatic progression, we deemed it appropriate to enhance these compounds with dual antitumor action by incorporating cytotoxic alkylating properties through the introduction of a nitrogen mustard moiety into their molecular structure. Based on one of the investigated ICADs, we successfully synthesized such a compound [56]—1-methyl-2-[bis(2-chloroethyl)aminomethyl]-3-carbethoxy-5-methoxy-6-bromoindole (Figure 1A; hereafter referred to as compound T1089). The presence of two electrophilic reactive sites (chloroethyl groups) in T1089 could potentially confer bifunctional alkylating properties similar to cyclophosphamide (CPA), which possesses a comparable reactive group (Figure 1B). The alkylating effect of bifunctional agents on DNA leads to difficult-to-repair damage (intrastrand or interstrand crosslinks and DNA strand breaks) and is characterized by high cytotoxic potential [17,19]. If the alkylating activity of T1089 is confirmed, this compound could be considered an AA with dual antitumor action: direct cytotoxic effects and indirect effects mediated by TLR-dependent stimulation of antitumor immunity. This may create prospects for its use in conventional chemotherapy and metronomic chemotherapy, particularly in combination with immune checkpoint inhibitors and antitumor vaccines [53,54].

This study provides a detailed description of the synthesis of T1089 and presents the results of the initial stage of pharmacological investigations, aimed at evaluating toxicological characteristics, clastogenic and mutagenic activity, cytotoxic potential in vitro, and antitumor effects in vivo under conventional chemotherapy regimens. For comparative assessment, CPA—a bifunctional AA widely used in conventional and metronomic chemotherapy—was employed as a reference compound.

## 2. Results

### 2.1. Synthesis of Compound T1089 and Its Physicochemical Properties

**Figure 2:**
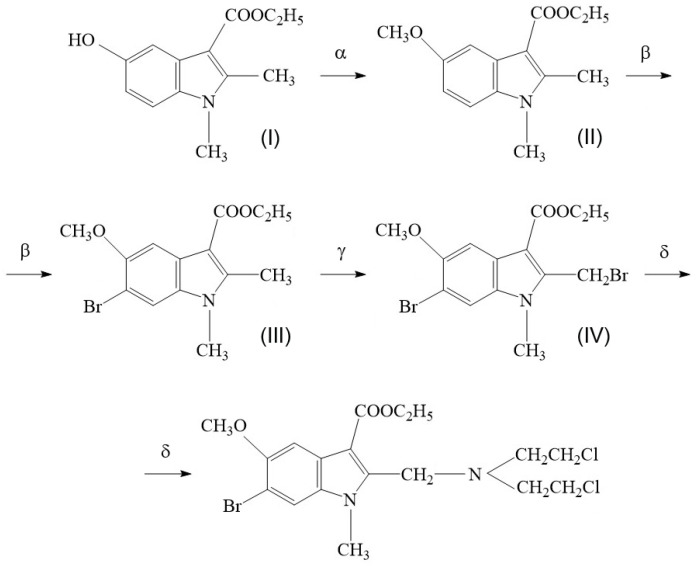
Synthesis scheme of compound T1089. Reagents, conditions and yields: α. (1) aq. NaOH, dioxane; (2) Me_2_SO_4_, 20 °C, 94%; β. *N*-bromosuccinimide, CCl_4_, boiling, 54%; γ. *N*-bromosuccinimide, (PhCOO)_2_, CCl_4_, irradiation (100 W bulb), boiling, 78%; δ. (1) dioxane, triethylamine; (2) di(2-chloroethyl)amine hydrochloride, dioxane, triethylamine, 60 °C, 67%.

The synthesis scheme for compound T1089 is illustrated in Figure 2. The starting material for the preparation of T1089 is a known derivative of indole-3-carbinol (Figure 2, Formula I)—1,2-dimethyl-3-carbethoxy-5-hydroxyindole (dimecarbin), which exhibits hypotensive activity [57]. The synthesis of T1089 was carried out in four stages, yielding three intermediate products (Figure 2, Formulas II–IV).

Stage 1—Preparation of 1,2-dimethyl-5-methoxy-3-ethoxycarbonylindole (Figure 2, Formula II).

To a solution of 0.02 M dimecarbin in 40 mL of dioxane, 40 mL of a 10% NaOH solution was added under stirring. Subsequently, 4 mL of dimethyl sulfate was added dropwise. The mixture was stirred for 1 h, during which a precipitate formed. The mixture was diluted with water, the precipitate was filtered off, and washed with water. Recrystallization was performed from ethanol. Yield: 4.65 g (94%). Mp 113–115 °C.

Stage 2—Preparation of 1,2-dimethyl-3-carbethoxy-5-methoxy-6-bromoindole (Figure 2, Formula III).

To a solution of 4.65 g (0.0188 M) of 1,2-dimethyl-5-methoxy-3-ethoxycarbonylindole in 75 mL of CCl_4_, 3.36 g (0.0188 M) of *N*-bromosuccinimide (NBS) was added. The mixture was refluxed for 4 h. Succinimide was filtered off while hot. The filtrate was cooled, filtered, washed with ethanol, and recrystallized from CCl_4_. Yield: 3.3 g (54%). Mp 156 °C.

Stage 3—Preparation of 1-methyl-2-bromomethyl-3-carbethoxy-5-methoxy-6-bromoindole (Figure 2, Formula IV).

A mixture of 3.3 g (0.01 M) of 1,2-dimethyl-3-carbethoxy-5-methoxy-6-bromoindole, 0.1 g (0.0004 M) of benzoyl peroxide, and 1.78 g (0.01 M) of *N*-bromosuccinimide in 50 mL of CCl_4_ was refluxed under illumination for 5 h. Succinimide was filtered off while hot. The filtrate was cooled, filtered, washed with ethanol, and recrystallized from CCl_4_. Yield: 3.16 g (78%). Mp 142 °C.

Stage 4—Final Stage.

The 1-methyl-2-bromomethyl-3-carbethoxy-5-methoxy-6-bromoindole obtained in Stage 3 was reacted with di(2-chloroethyl)amine hydrochloride, which was preliminarily prepared by the reaction of diethanolamine with thionyl chloride in chloroform. To a solution of 0.64 g (0.005 M) of di(2-chloroethyl)amine hydrochloride in 30 mL of dioxane, 0.7 mL of triethylamine (TEA) was added. Separately, 2.05 g (0.005 M) of 1-methyl-2-bromomethyl-3-carbethoxy-5-methoxy-6-bromoindole was dissolved in 30 mL of dioxane and 0.7 mL of TEA. The solutions were combined and heated at 60 °C. The precipitated TEA·HCl and TEA·HBr were filtered off and washed with hot dioxane. The filtrate was evaporated, and the residue was triturated with 5–7 drops of water. Recrystallization was performed from isopropanol. Yield: 1.57 g (67%). Mp 99–100 °C.

**Figure 3:**
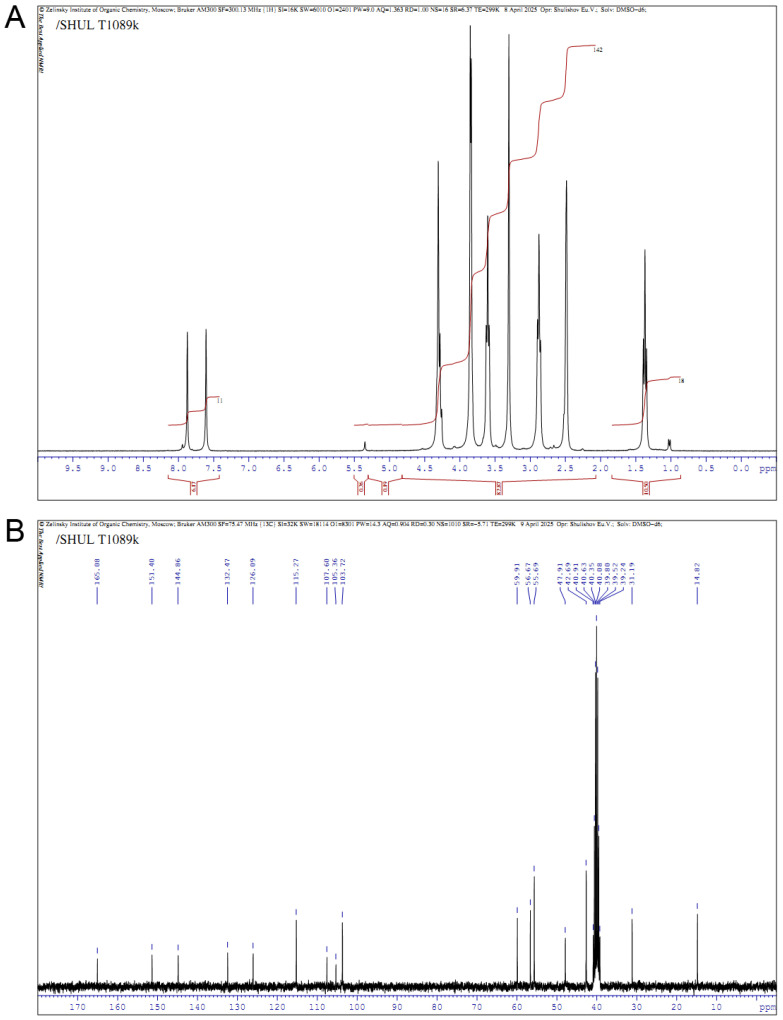
NMR spectra of compound T1089. (**A**)—on ^1^H nuclei; (**B**)—on ^13^C nuclei. Descriptions of the spectra are given in the text.

The structure of the obtained compound T1089 and the acceptable purity of the substance were confirmed by the correspondence of the ^1^H and ^13^C NMR spectra to the structure of 1-methyl-2-[bis(2-chloroethyl)aminomethyl]-3-carbethoxy-5-methoxy-6-bromoindole—the presence of all expected signals with their typical position and manifestation, and the absence of additional signals (Figure 3).

NMR spectrum ^1^H (300.1 MHz, DMSO-*d*_6_): δ 1.39 (t, 3 H, *J* = 7.0 Hz, CH_3_), 2.89 (t, 4 H, *J* = 6.6 Hz, 2 NCH_2_), 3.61 (t, 4 H, *J* = 6.6 Hz, 2CH_2_Cl), 3.84 and 3.87 (both s, 2 × 3 H, NCH_3_ and OCH_3_), 4.29 (s, 2 H, NCH_2_), 4.30 (q, 2 H, *J* = 7.0 Hz, CH_2_O), 7.60 (s, 1 H, H^4^), 7.88 (s, 1 H, H^7^).

NMR spectrum ^13^C (75.5 MHz, DMSO-*d*_6_): δ 14.8 (CH_3_), 31.2 (NCH_3_), 42.7 (2 CH_2_Cl), 47.9 (NCH_2_), 55.7 (2 NCH_2_), 56.7 (OCH_3_), 59.9 (CH_2_O), 103.7 (C^4^), 105.4 and 107.6 (C^3^, C^6^), 115.3 (C^7^), 126.1 (C^3a^), 132.5 (C^7a^), 144.9 (C^2^), 151.4 (C^5^), 165.1 (C=O).

The structure of T1089 was also confirmed by high-resolution mass spectrometry (HRMS) and elemental analysis (EA) of C, H and N. HRMS: [M+H]^+^ calculated for C_18_H_24_BrCl_2_N_2_O_3_ 467.2096; found *m*/*z* 467.2092 (Figure S1). EA: calculated (%) for C_18_H_23_BrCl_2_N_2_O_3_ C 46.30, H 4.96, N 5.90; found (%) C 46.33, H 5.05, N 5.81.

Chemical formula T1089: C_18_H_23_BrCl_2_N_2_O_3_. Molecular weight T1089: 466.2 g/mol.

The substance of compound T1089 is a powder of non-uniform flakes of pale yellow color, insoluble in water, well soluble in alcohols, DMSO, dioxane and chloroform.

### 2.2. Toxicological Properties of Compound T1089

The toxicological characteristics of T1089 were studied in outbred male ICR (CD-1) mice using an acute toxicity test following a single intraperitoneal (i.p.) administration at doses ranging from 180 to 220 mg/kg. The mice tolerated T1089 satisfactorily at the tested doses, with no significant changes in behavior or signs of acute intoxication observed. However, at doses of 190 mg/kg and higher, a decrease in body weight, increasing hypoactivity, and delayed mortality were noted in some mice by days 4–7 post-administration.

During the 14-day observation period, the mortality rate in the experimental groups exhibited a dose-dependent pattern:

0.0% (0/5) at 180 mg/kg
12.5% (1/8) at 190 mg/kg
50.0% (3/6) at 200 mg/kg
75.0% (6/8) at 210 mg/kg
100.0% (5/5) at 220 mg/kg

Probit analysis of these data yielded the following standard toxicity parameters:

LD_10_: 188 mg/kg
LD_16_: 191 mg/kg
LD_50_: 202 ± 6 mg/kg
LD_84_: 213 mg/kg

Based on these values, compound T1089 can be classified as a moderately toxic substance (Hazard Class 3) [58].

### 2.3. Cytotoxic Activity of Cyclophosphamide and T1089 In Vitro

**Figure 4:**
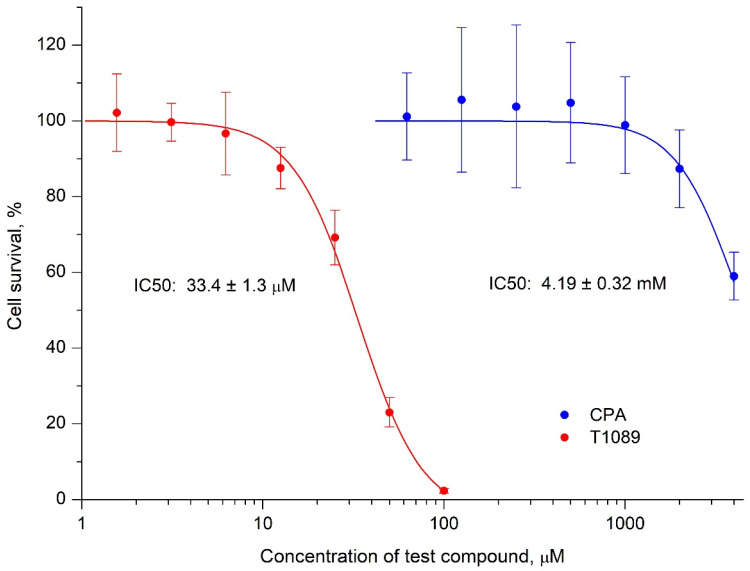
Cytotoxic effect of compound T1089 and cyclophosphamide (CPA) in vitro on human lung carcinoma cells A549 according to MTT test data. Graphic symbols—survival of A549 cells (M ± SD; *n* = 12) after 72 h of growth in the presence of different concentrations of test compounds. Curves are lines of logistic regressions “concentration (μM)—survival (%)”, calculated from experimental data. IC_50_ values were estimated from the regression parameters.

According to the MTT assay data, the cytotoxic effects of CPA and compound T1089 on human lung carcinoma A549 cells qualitatively differed (Figure 4; Tables S1 and S2). The in vitro effect of CPA on A549 cells was weakly toxic, and the observed cytotoxic effects of CPA were likely nonspecific, reflecting the general toxic properties of this compound. Significant cytotoxicity was only observed at high concentrations exceeding 2 mM (IC_50_ = 4.2 ± 0.3 mM), corresponding to doses above the median lethal dose (LD_50_ = 140 mg/kg [59]).

In contrast, the in vitro effect of compound T1089 on A549 cells was 125 times more cytotoxic. Significant cytotoxic effects of T1089 were observed even at low concentrations (15–50 μM; IC_50_ = 33.4 ± 1.3 μM), corresponding to doses that are safe in terms of lethality (7–23 mg/kg, 1/30–1/9 of LD_50_).

### 2.4. Clastogenic (Mutagenic) Activity of Cyclophosphamide and T1089 In Vivo

**Figure 5:**
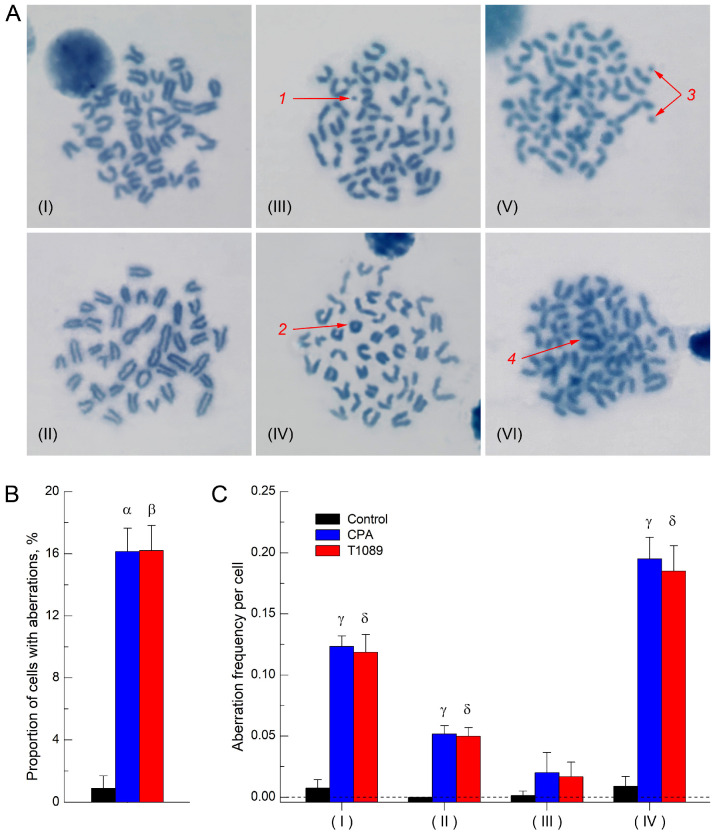
The effect of cyclophosphamide and compound T1089 on structural mutagenesis in bone marrow cells of female F_1_ (C57Bl/6j × CBA) mice according to the chromosome aberration test. (**A**) Pictures of metaphases of bone marrow cells of mice. Stained with Giemsa dye, ×1250. (**I**,**II**)–cells with a normal karyotype, represented by 40 acrocentric chromosomes. (**III**–**VI**)–cells with chromosomal structural changes: 1—single fragment; 2—ring chromosome; 3—paired fragment; 4—dicentric chromosome. (**B**,**C**) Structural mutagenesis indices in mice of experimental groups (6 animals in each group, graphical deviations correspond to SD). (**B**) Proportion of bone marrow cells with chromosome aberrations. Symbols—significant differences in this indicator: α—in control mice vs. CPA-treated mice (*p* = 0.01166); β—in control mice vs. T1089-treated mice (*p* = 0.01166). (**C**) Frequency of different types of chromosome aberrations: (**I**)—breaks, single and paired acentric fragments; (**II**)—ring, dicentric and metacentric chromosomes; (**III**)—achromatic gaps; (**IV**)—all chromosome aberrations. Symbols—significant differences in these indicators: γ—in control mice vs. CPA-treated mice (*p* = 0.01166; *p* = 0.00615; *p* = 0.01166); δ—in control mice vs. T1089-treated mice (*p* = 0.01166; *p* = 0.00419; *p* = 0.01166).

A comparative study of the clastogenic activity (ability to induce DNA strand breaks) of CPA and compound T1089 was conducted in female F_1_ (CBA × C57BL6j) mice using the chromosome aberration test in bone marrow cells [60]. It was found that treatment with 0.9% sodium chloride did not significantly affect structural mutagenesis in the bone marrow cells of mice. At the first mitosis stage after treatment, the frequency of karyocytes with aberrations, represented by rare chromatid fragments, in control mice was 0.9% (Figure 5), which corresponds to the level of spontaneous structural mutagenesis typical for these animals [61,62].

In contrast, a single i.p. injection of CPA and T1089 at an equimolar dose of 0.064 mmol/kg (16.9 and 30.0 mg/kg, respectively) resulted in a statistically significant (*p* = 0.0042–0.0117), qualitatively and quantitatively similar stimulation of structural mutagenesis associated with DNA strand break formation [63,64]. At the first mitosis stage after exposure, the frequency of karyocytes with aberrations in CPA-treated and T1089-treated mice increased to 16.2–16.5%, primarily due to a rise in chromatid breaks and fragments and, to a lesser extent, an increase in the frequency of exchange aberrations (Figure 5; Table S3). At the same time, no statistically significant increase in the frequency of achromatic gaps (which do not represent true DNA breaks [63]) was observed in the karyocytes of mice treated with CPA and T1089.

### 2.5. In Vivo Antitumor Effect of Cyclophosphamide and T1089 on Solid Ehrlich Carcinoma

A comparative study of the antitumor activity of CPA and T1089 in vivo was conducted on Ehrlich solid carcinoma (ESC) in female F_1_ (CBA × C57BL6j) mice. The test compounds were administered intraperitoneally (i.p.) seven times at 48 h intervals at a relatively safe dose of 33 mg/kg (corresponding to 1/4 of the LD_50_ for CPA and 1/6 of the LD_50_ for T1089).

**Figure 6:**
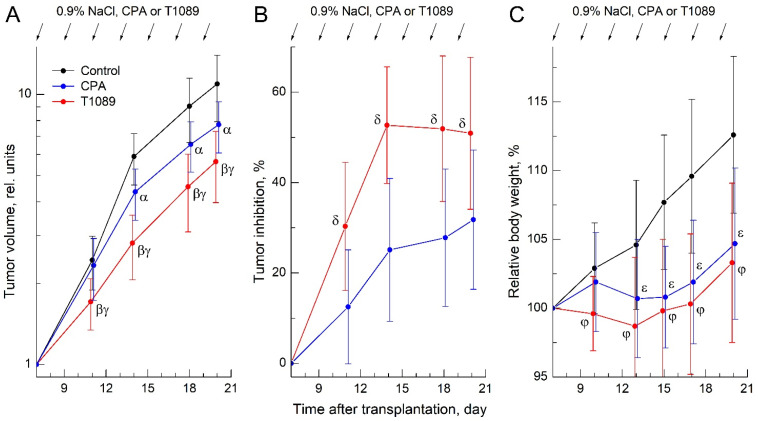
Antitumor and toxic effects of cyclophosphamide (CPA) and compound T1089 with chronic i.p. administration at a dose of 33 mg/kg (with an interval of 48 h) in a model of Ehrlich solid carcinoma (ESC) in female F_1_ (C57Bl/6j × CBA) mice. (**A**) Growth curves of ESC in experimental groups of animals. The tumor volume (TV) values for each animal were normalized to the initial neoplasia volume on the day of treatment initiation (7th day after transplantation). Graphic deviations correspond to SD (*n* = 19–21 per point). Symbols—significant TV differences: α—in control mice vs. CPA-treated mice (*p* = 0.00034; *p* = 0.00045; *p* = 0.00013); β—in control mice vs. T1089-treated mice (*p* = 0.00008; *p* < 0.00001; *p* < 0.00001; *p* = 0.00001); γ—in CPA-treated mice vs. T1089-treated mice (*p* = 0.00050; *p* = 0.00002; *p* = 0.00017; *p* = 0.03487). (**B**) Dynamics of the tumor inhibition (TI) index in mice receiving experimental therapy. Graphic deviations correspond to SD (*n* = 20–21 per point). Symbols δ—significant TI differences in CPA-treated mice vs. T1089-treated mice (*p* = 0.00029; *p* = 0.00001; *p* = 0.00009; *p* = 0.00734). (**C**) Dynamics of relative body weight (BW) in mice of experimental groups. Graphic deviations correspond to SD (*n* = 19–21 per point). Symbols—significant BW differences: ε—in control mice vs. CPA-treated mice (*p* = 0.00993; *p* = 0.00011; *p* = 0.00015; *p* = 0.00074); φ—in control mice vs. T1089-treated mice (*p* = 0.01728; *p* = 0.00162; *p* = 0.00007; *p* = 0.00008; *p* = 0.00054).

The obtained data indicated high resistance of ESC to CPA at the administered dose. The antitumor effect of CPA increased gradually over the course of treatment, reaching a significant level only after four injections. By the end of the observation period, following seven administrations, the effect remained moderate, with the tumor inhibition (TI) index not exceeding 30% (Figure 6A,B; Tables S5 and S6).

In contrast, ESC exhibited substantially higher sensitivity to T1089. The antitumor effect of T1089 upon repeated administration also displayed a cumulative and progressive nature, but the rate of development and magnitude of the effect were significantly greater. A statistically significant effect of T1089 was observed as early as after two injections, reached its maximum (TI index—53%) after four administrations, and remained at this high level until the end of the experiment (Figure 6A,B; Tables S5 and S6).

However, alongside the antitumor effects, this experiment also revealed negative, toxic effects of CPA and T1089. According to clinical observations, mice bearing ESC tolerated the seven i.p. administrations of CPA and T1089 at the tested dose satisfactorily. No signs of acute intoxication, changes in behavioral responses or feeding activity, or animal mortality were observed. However, body weight (BW) monitoring indicated the development of a significant deficit (8–10% BW) in CPA-treated and T1089-treated mice compared to control animals (Figure 6C; Table S7). Moreover, this negative effect developed slightly faster in mice treated with T1089.

### 2.6. In Vivo Antitumor Effect of T1089 on Melanoma B-16

To more objectively evaluate the antitumor potential of T1089 in vivo and determine its optimal therapeutic dose, a study was conducted to assess the effects of this compound on B-16 melanoma in female C57BL6j mice. The compound was administered intraperitoneally (i.p.) six times at 48 h intervals at a dose of 28 mg/kg (1/7 of the LD_50_).

**Figure 7:**
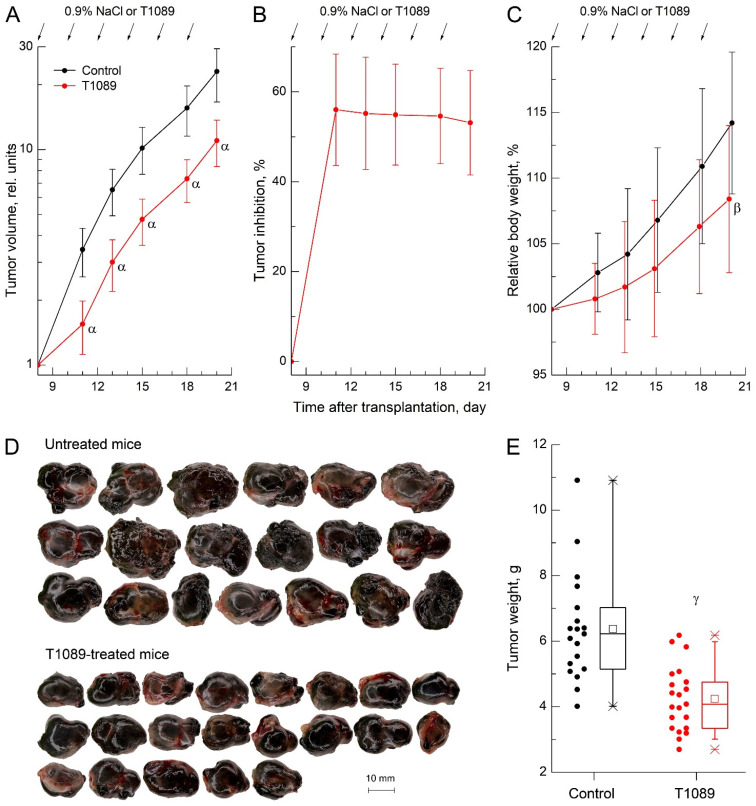
Antitumor and toxic effects of compound T1089 with chronic i.p. administration at a dose of 28 mg/kg (with an interval of 48 h) in a model of melanoma B-16 in female C57Bl/6j mice. (**A**) Growth curves of melanoma in experimental groups of animals. The tumor volume (TV) values for each animal were normalized to the initial neoplasia volume on the day of treatment initiation (8th day after transplantation). Graphic deviations correspond to SD (*n* = 19–21 per point). Symbols α—significant TV differences in control mice vs. T1089-treated mice (*p* < 0.00001; *p* < 0.00001; *p* < 0.00001; *p* = 0.00001; *p* = 0.00003). (**B**) Dynamics of the tumor inhibition index in mice receiving experimental therapy. Graphic deviations correspond to SD (*n* = 19–21 per point). (**C**) Dynamics of relative body weight (BW) in mice of experimental groups. Graphic deviations correspond to SD (*n* = 19–21 per point). Symbol β—a significant BW difference in control mice vs. T1089-treated mice (*p* = 0.02174). (**D**) Appearance of melanoma B-16 tumor nodes in control and T1089-treated mice on the 20th day after transplantation. (**E**) Weight of melanoma tumor nodes in control and T1089-treated mice on the 20th day after transplantation. Symbol γ—a significant tumor weight difference in control mice vs. T1089-treated mice (*p* = 0.00002).

The results of this experiment demonstrated that B-16 melanoma was highly sensitive to T1089 at the administered dose. After just two injections, the antitumor effect of T1089 reached its maximum (TI index—56%), and the subsequent four injections maintained a high level of efficacy (TI index—52–55%) until the end of the observation period (Figure 7A,B; Tables S9 and S10). The pronounced antitumor activity of T1089 against B-16 melanoma was further supported by terminal tumor assessments. Melanoma nodules in control mice were visibly larger and exhibited a significantly higher mass compared to those in T1089-treated mice (Figure 7D,E).

An important finding of this experiment was that at this lower dose, T1089 produced such a significant antitumor effect with minimal toxicity. In this case, the body weight (BW) deficit in T1089-treated mice compared to controls was limited and reached a significant level only at the final observation time point (Figure 7C). This difference may not have been due to the toxic effects of T1089 but rather to the ~2 g disparity in average melanoma tumor mass between the groups.

## 3. Discussion

The compound we synthesized, T1089 (Figure 1A), is a new *N*-mustard based on an indole-3-carboxylic acid derivative. Notably, the indole-3-carboxylic acid derivative used as the basis for T1089 is a TLR agonist [51] capable of activating an antitumor immune response [55].

Toxicological studies have established that T1089 is a relatively safe compound, classified as hazard class 3 [58] (moderately toxic substances). Its acute toxicity indices, LD_16_ and LD_50_, in mice upon intraperitoneal (i.p.) administration are 191 and 202 mg/kg, respectively. By this measure, T1089 is slightly safer than cyclophosphamide (CPA), which has an LD_50_ of 140 mg/kg [59].

The presence of two reactive sites (chloroethyl groups) in the structure of T1089 confers upon this compound the properties of bifunctional alkylating agents (AAs), similar to CPA, which has an analogous reactive group. The alkylating effect of such agents on DNA leads to the formation of difficult-to-repair damage (intra- or interstrand cross-links and DNA strand breaks), accompanied by the stimulation of structural mutagenesis and a pronounced cytotoxic effect [17,19]. The alkylating activity and clastogenic potential of T1089 were demonstrated by the qualitative and quantitative similarity of the effects of CPA and T1089 in the mammalian bone marrow chromosome aberration test (Figure 5; Table S3). A single i.p. administration of CPA and T1089 to mice at an equimolar dose of 0.064 mmol/kg resulted in a similar, statistically significant (*p* = 0.0042–0.0117) stimulation of structural mutagenesis associated with the formation of DNA strand breaks [63,64]. At the first mitosis stage after exposure, the frequency of karyocytes with aberrations in CPA-treated and T1089-treated mice increased 20-fold compared to control animals, primarily due to an increase in chromatid breaks and fragments and, to a lesser extent, an increase in the frequency of exchange-type aberrations. At the same time, no significant increase in the frequency of achromatic gaps—which do not correspond to true DNA breaks [63]—was observed upon exposure to CPA or T1089.

At the same time, the data from studies on the cytotoxic effects of CPA and T1089 in vitro indicated that the mechanism of alkylating action likely differs significantly between these compounds (Figure 4; Tables S1 and S2). It is known that the release of the alkylating metabolite (*N*-mustard) from CPA occurs in two stages: the first involves hepatic microsomal enzymes (mainly cytochrome P450), and the second involves cellular phosphatases [17,19]. In this regard, under in vitro conditions (in the absence of microsomal enzymes), CPA exhibits almost no cytotoxic effects. This was also confirmed by our MTT assay results, where weak cytotoxic effects of CPA were observed only at concentrations exceeding 2 mM (IC_50_—4.2 ± 0.3 mM), corresponding to supralethal doses in vivo (>4 LD_50_). The mechanisms of the alkylating action of T1089 have not yet been studied, but this process is presumably simpler than that of CPA, as this compound exhibited pronounced cytotoxicity in vitro. According to the MTT assay, significant cytotoxic effects of T1089 were observed even at low concentrations of 15–50 µM (IC_50_—33.4 ± 1.3 µM), corresponding to safe in vivo doses of 7–23 mg/kg (1/30–1/9 LD_50_).

Significant differences in the effects of CPA and T1089 were also observed in the study of antitumor activity in vivo on the model of conventional ESC chemotherapy in mice (Figure 6; Tables S5–S7). The agents were administered intraperitoneally (i.p.) seven times at 48 h intervals at a dose of 33 mg/kg (1/4 LD_50_ for CPA, 1/6 LD_50_ for T1089). At this dose and route of administration, neither CPA nor T1089 caused acute intoxication or animal death. However, this chemotherapy regimen with these agents was accompanied by increasing toxic effects, manifested by the cumulative development of significant body weight loss in CPA-treated and T1089-treated mice.

Despite the relatively “harsh” chemotherapy regimen, ESC exhibited significant resistance to CPA. The antitumor effect of CPA increased slowly over the course of treatment, reaching a significant level only after four injections, and by the end of observations—after seven administrations—remained moderate, with the TI index not exceeding 30%.

In contrast, ESC was substantially more sensitive to T1089 under the same chemotherapy conditions, and at all observation time points, the effects of T1089 significantly exceeded those of CPA. The dynamics of T1089′s antitumor effect also followed a cumulative pattern, but the rate of development and magnitude of the effect were qualitatively higher. A significant effect of T1089 was achieved after just two injections, with the maximum effect (TI index—53%) observed after four administrations. This high level of efficacy was maintained until the end of the experiment.

The high antitumor efficacy of T1089 in vivo was also confirmed in studies using the B-16 melanoma chemotherapy model in mice (Figure 7; Tables S9–S12). When administered intraperitoneally (i.p.) six times at 48 h intervals at a dose of 28 mg/kg (1/7 of the LD_50_), chemotherapy with T1089 proceeded with minimal signs of toxicity. Body weight loss in T1089-treated mice was limited and reached a significant level only at the final observation time point, most likely due not to the toxic effects of T1089 but to differences in melanoma tumor mass between the groups.

At the same time, B-16 melanoma remained highly sensitive to the action of T1089 even in this milder chemotherapy regimen. After two injections, the antitumor effect of T1089 reached its maximum development (TI index—56%), and the subsequent four injections maintained a consistently high level of efficacy (TI index—52–55%) until the end of the observation period.

Thus, the data obtained during the initial stage of pharmacological studies of T1089 indicate that this compound possesses alkylating activity characteristic of bifunctional agents, which is accompanied by high cytotoxic activity in vitro and pronounced antitumor effects in vivo in animal tumor models of conventional chemotherapy (at high doses of the agent). At the same time, given that the indole-3-carboxylic acid derivative used as the basis for T1089 is a TLR agonist capable of activating an antitumor immune response, T1089 can be considered an alkylating agent with dual antitumor action. Overall, this highlights the need for further studies of this agent in conventional chemotherapy and metronomic chemotherapy, particularly in combinations with immune checkpoint blockers and antitumor vaccines.

## 4. Materials and Methods

### 4.1. T1089 and Other Compounds and Solutions

The investigated compound T1089 was synthesized in the Laboratory of Radiation Pharmacology at A. Tsyb MRRC. The synthetic methodology for T1089 was previously reported in a patent application [56] and is further detailed in the Results section of this article. Structural confirmation of T1089 was achieved through NMR spectroscopy, high-resolution mass spectrometry (HRMS), and elemental analysis.

^1^H (300.1 MHz) and ^13^C (75.5 MHz) NMR spectra were recorded in DMSO-*d*_6_ containing 0.05% tetramethylsilane (TMS) as an internal standard, using a Bruker Avance AV 300 Fourier spectrometer (Karlsruhe, Germany). High-resolution mass spectra were acquired using an ESI-QTOF Impact mass spectrometer (Bruker Daltonik, Bremen, Germany). Elemental analysis (C, H, N) was performed on a Carlo Erba EA 1108 elemental analyzer (Carlo Erba Instruments, Milan, Italy). Purity assessment was conducted via thin-layer chromatography (TLC) and melting point determination. TLC was carried out on Silufol UV-254 plates (Kavalier, Czech Republic) using a mobile phase of benzene–ethanol–triethylamine (9:1:0.1). The melting point was measured using an automatic PTP-M heating unit (LOIP Ltd., Saint Petersburg, Russia).

T1089 is a pale-yellow, flaky powder, insoluble in water but readily soluble in alcohols, DMSO, dioxane, and chloroform. For in vivo studies (acute toxicity, chromosome aberration assay, and antitumor efficacy in solid tumor models), T1089 was administered intraperitoneally (i.p.) as an aqueous suspension prepared ex tempore using sterile water for injection (Grotex, Saint Petersburg, Russia) with 0.5% hypromellose (Nippon Soda Co., Tokyo, Japan) as a suspending agent. For in vitro cytotoxicity assays, T1089 was dissolved in 1.0% DMSO (PanEco Ltd., Kaluga, Russia).

In most experiments, cyclophosphamide (CPA) served as the reference compound. CPA was obtained as a pharmacopoeial-grade powder (Endoxan; Baxter, Unterschleissheim, Germany) for intravenous administration and was dissolved in either sterile water for injection or deionized water. In in vivo studies, control (untreated) animals received an equivalent volume of 0.9% sodium chloride (Dalchimpharm JSC, Khabarovsk, Russia) administered i.p.

### 4.2. In Vitro Cytotoxic Activity Studies

Comparative in vitro study of the cytotoxic activity of CPA and compound T1089 was performed using the MTT assay on human lung carcinoma A549 cells. CPA was tested at concentrations ranging from 62.5 to 4000 μM, while T1089 was evaluated at concentrations of 1.56 to 100 μM. The concentration ranges were determined based on preliminary MTT assay data for these compounds.

T1089 was dissolved in a 1% DMSO solution (PanEco Ltd., Kaluga, Russia) prepared in DMEM medium (PanEco) supplemented with fetal bovine serum (PanEco). The final DMSO concentrations did not exceed 0.5% and were non-toxic to the cells. CPA was dissolved in deionized water (PanEco).

The test compounds were added to the culture medium, followed by cell incubation, MTT dye (PanEco) addition, formazan extraction, spectrophotometric measurements, and quantitative data analysis, as previously described in our study [65]. The IC_50_ values were determined using logistic regression models fitted to the experimental data.

### 4.3. Animals

The in vivo studies were conducted on 31 male outbred ICR (CD-1) mice (age 2.5–3 months, body weight 22–25 g), 81 female F_1_ (CBA × C57BL6j) mice (age 2–2.5 months, body weight 19–22 g), and 42 female C57BL6j mice (age 2–2.5 months, body weight 18–21 g). The mice were obtained from the animal breeding facility of the National Center for Biomedical Technologies (NCBT) of the FMBA of Russia, underwent quarantine, and were housed in the MRRC vivarium under natural lighting conditions, with 10 air changes per hour, at a temperature of 18–20 °C and relative humidity of 40–70%. They had ad libitum access to water and rodent feed PK-120-1 (Laboratorkorm Ltd., Moscow, Russia).

Animal experiments were approved by the Ethics Committee of MRRC and performed in accordance with standard operating procedures compliant with the requirements of the European Convention ETS/STE No. 123. Scheduled euthanasia of mice was carried out using a CO_2_ euthanasia device AVTech (Inpren Ltd., Moscow, Russia).

### 4.4. Toxicological Studies of Compound T1089

Toxicological studies of T1089 were conducted on 31 male outbred ICR (CD-1) mice using the Litchfield and Wilcoxon method in an acute toxicity test following a single intraperitoneal (i.p.) administration [66]. The animals were divided into 5 groups (5–8 mice per group), which received T1089 at doses of 180, 190, 200, 210, and 220 mg/kg (10.0 mL/kg of a 1.8–2.2% suspension). The tested dose range was determined based on a preliminary estimation of the LD_50_ (200 mg/kg) obtained using the Deichmann and LeBlanc method [66]. Subsequently, over the following 14 days, the clinical manifestations of T1089 toxic effects were evaluated, and the time to death was recorded. At the end of the observation period, a probit-logarithmic regression analysis of “dose (mg/kg)–mortality (%)” was performed based on the 14-day lethality data, from which standard acute toxicity parameters (LD_10_, LD_16_, LD_50_, and LD_84_) were derived.

### 4.5. Study of Clastogenic (Mutagenic) Activity of Cyclophosphamide and T1089

The comparative investigation of the clastogenic effect (ability to induce DNA strand breaks) of cyclophosphamide (CPA) and compound T1089 was conducted using 18 female F_1_ (CBA × C57BL6j) mice in a standard mammalian bone marrow chromosome aberration assay [60]. The animals were divided into three groups, with six mice per group.

The first group received a single intraperitoneal (i.p.) injection of 0.9% sodium chloride at a dose of 10.0 mL/kg. The second group was administered CPA at a dose of 0.064 mmol/kg (16.9 mg/kg; 10.0 mL/kg of a 0.17% solution) via the same route. The third group received T1089 at an equimolar dose of 0.064 mmol/kg (30.0 mg/kg; 10.0 mL/kg of a 0.3% suspension).

Twenty-two hours after treatment, all animals were injected intraperitoneally (i.p.) with colchicine (PanEco) at a dose of 4 mg/kg. After 24 h, the mice were euthanized via CO_2_ asphyxiation. Bone marrow extraction, hypotonic treatment, suspension preparation and fixation, slide preparation, Giemsa staining, and microscopic analysis were performed according to established guidelines [60,67].

For each animal, 110–130 well-spread metaphases containing at least 40 centromeres were analyzed under high magnification using a Leica DM 1000 microscope (Leica Microsystems CMS GmbH, Wetzlar, Germany). Metaphases with excessively scattered chromosomes or those with very short and/or misaligned chromatids were excluded. The following aberrations were recorded per metaphase: achromatic gaps, chromatid breaks (with or without accompanying fragments), chromatid fragments, chromosome fragments, chromatid exchanges, and chromosome exchanges.

For statistical analysis, the minimal observational units were the frequency of aberrant cells and the frequencies of different aberration types per individual mouse.

### 4.6. Studies of the Antitumor Activity of Cyclophosphamide and T1089 In Vivo

Studies on antitumor activity in vivo were conducted on two tumor models: solid Ehrlich carcinoma (ESC) in female F_1_ (CBA × C57BL6j) mice and B-16 melanoma in female C57BL6j mice.

ESC was transplanted into 63 female F_1_ (CBA × C57BL6j) mice by subcutaneous injection into the lateral surface of the right thigh with 2.5 × 10^6^ tumor cells in 0.1 mL of a DMEM-based suspension. The animals were then divided into three groups—a control group and two experimental groups (21 mice each). Treatment was initiated on day 7 after tumor transplantation. Mice in the experimental groups received seven intraperitoneal (i.p.) injections (at 48 h intervals) of CPA and T1089 at a dose of 33 mg/kg (10.0 mL/kg of a 0.33% CPA solution or 0.33% T1089 suspension) from day 7 to day 19 of tumor growth. Mice in the control group were administered 0.9% sodium chloride i.p. at a dose of 10.0 mL/kg according to the same schedule. Potential toxic effects of CPA and T1089 were assessed through daily animal observations and body weight dynamics. The influence of CPA and T1089 on tumor growth was evaluated by monitoring ESC progression in the groups. For this purpose, from day 7 to day 20 of ESC growth, the orthogonal dimensions of the tumor nodes were measured 2–3 times per week in all animals using a digital caliper DC-1-125 (SPE CIP, Moscow, Russia). Absolute and relative tumor volumes (TV) and tumor inhibition (TI) indices were calculated using the methods described in our previous work [68]. The antitumor effects of CPA and T1089 were evaluated and compared through intergroup statistical analyses of TV and TI indices at different observation time points.

B-16 melanoma was transplanted into 42 female C57BL6j mice by subcutaneous injection into the lateral surface of the right thigh with 10^6^ tumor cells in 0.1 mL of a DMEM-based suspension. The animals were then divided into two groups—a control group and an experimental group (21 mice each). Treatment was initiated on day 8 after tumor transplantation. Mice in the experimental group received six i.p. injections (at 48 h intervals) of T1089 at a dose of 28 mg/kg (10.0 mL/kg of a 0.28% suspension) from day 8 to day 18 of tumor growth. Mice in the control group were administered 0.9% sodium chloride i.p. at a dose of 10.0 mL/kg following the same schedule. Potential toxic and antitumor effects of T1089 were assessed in this experiment using the same methods as in the ESC study. Additionally, at the end of the observation period (on day 20 of tumor growth), melanoma nodes were excised from all mice, and macroscopic and massometric comparisons were performed between the groups.

### 4.7. Statistical Analysis

For all controlled quantitative indicators, the calculation of variation parameters was performed, and their values are presented, including graphically, as M ± SD. The statistical significance of intergroup differences was assessed using nonparametric methods: for pairwise comparison—with the Mann–Whitney U test; for multiple comparisons—with the Kruskal–Wallis rank ANOVA test, followed by post hoc analysis using the Mann–Whitney U test with Holm–Bonferroni corrections [69]. Differences were considered statistically significant at the 5% significance level. Statistical calculations and regression modeling were performed using the software packages Statistica 10.0 (StatSoft Inc., Tulsa, OK, USA) and BioStat 7.3 (AnalystSoft Inc., Alexandria, CA, USA).

## Data Availability

The original contributions presented in this study are included in the article and Supplementary Materials. Further inquiries can be directed to the corresponding author.

## Supplementary Materials

The following supporting information can be downloaded at: https://www.mdpi.com/article/10.3390/molecules30183710/s1, Figure S1: High-resolution mass spectrometry of T1089; Table S1: Initial photometric data; Table S2: A549 cell survival, IC50 value estimates; Table S3: Structural mutagenesis indices in bone marrow cells of control, CPA-treated and T1089-treated mice; Table S4: Absolute growth dynamics of solid Ehrlich carcinoma in groups of mice; Table S5: Relative growth dynamics of solid Ehrlich carcinoma in groups of mice; Table S6: Tumor growth inhibition in treated mice; Table S7: Dynamics of relative body weight of mice; Table S8: Absolute growth dynamics of melanoma B-16 in groups of mice; Table S9: Relative growth dynamics of melanoma B-16 in groups of mice; Table S10: Tumor growth inhibition in treated mice; Table S11: Melanoma nodule weight on day 20th after transplantation; Table S12: Dynamics of relative body weight of mice.

## Abbreviations

AAs—alkylating agents; ATE—antitumor effect; BMCA—bone marrow chromosome aberration, BW—body weight; CCT—conventional chemotherapy; CPA—cyclophosphamide; ESC—Ehrlich solid carcinoma; IARC—International Agency for Research on Cancer; ICAD—indole-3-carboxylic acid derivative, MTD—maximum tolerated dose; TI—tumor inhibition; TV—tumor volume.
