# Supplementary material for: Synthesis, Clastogenic and Cytotoxic Potential, and In Vivo Antitumor Activity of a Novel N-Mustard Based on Indole-3-carboxylic Acid Derivative
Source: Molecules. 2025 Sep 12;30(18):3710. doi: 10.3390/molecules30183710 (PMC12472313; doi:10.3390/molecules30183710)

Display Report

Analysis Info

Analysis Name	D:\Data\Kolotyrkina\2025\Shulishov\0409017.d	Acquisition Date	09.04.2025 14:37:52
Method	tune_low.m	Operator	BDAL@DE
Sample Name	/SHUL T-1089k	Instrument / Ser#	micrOTOF 10248
Comment	C19H26Cl2N2O4 clb added CH3CN		

Acquisition Parameter

Source Type	ESI	Ion Polarity	Positive	Set Nebulizer	0.4 Bar
Focus	Not active			Set Dry Heater	180 °C
Scan Begin	50 m/z	Set Capillary	4500 V	Set Dry Gas	4.0 l/min
Scan End	3000 m/z	Set End Plate Offset	-500 V	Set Divert Valve	Waste

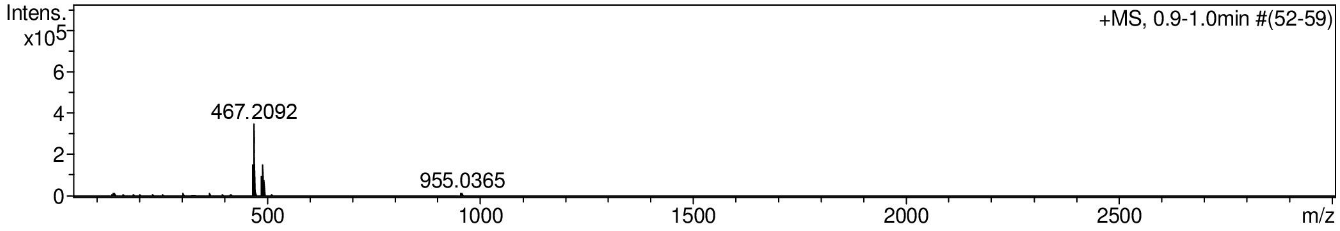

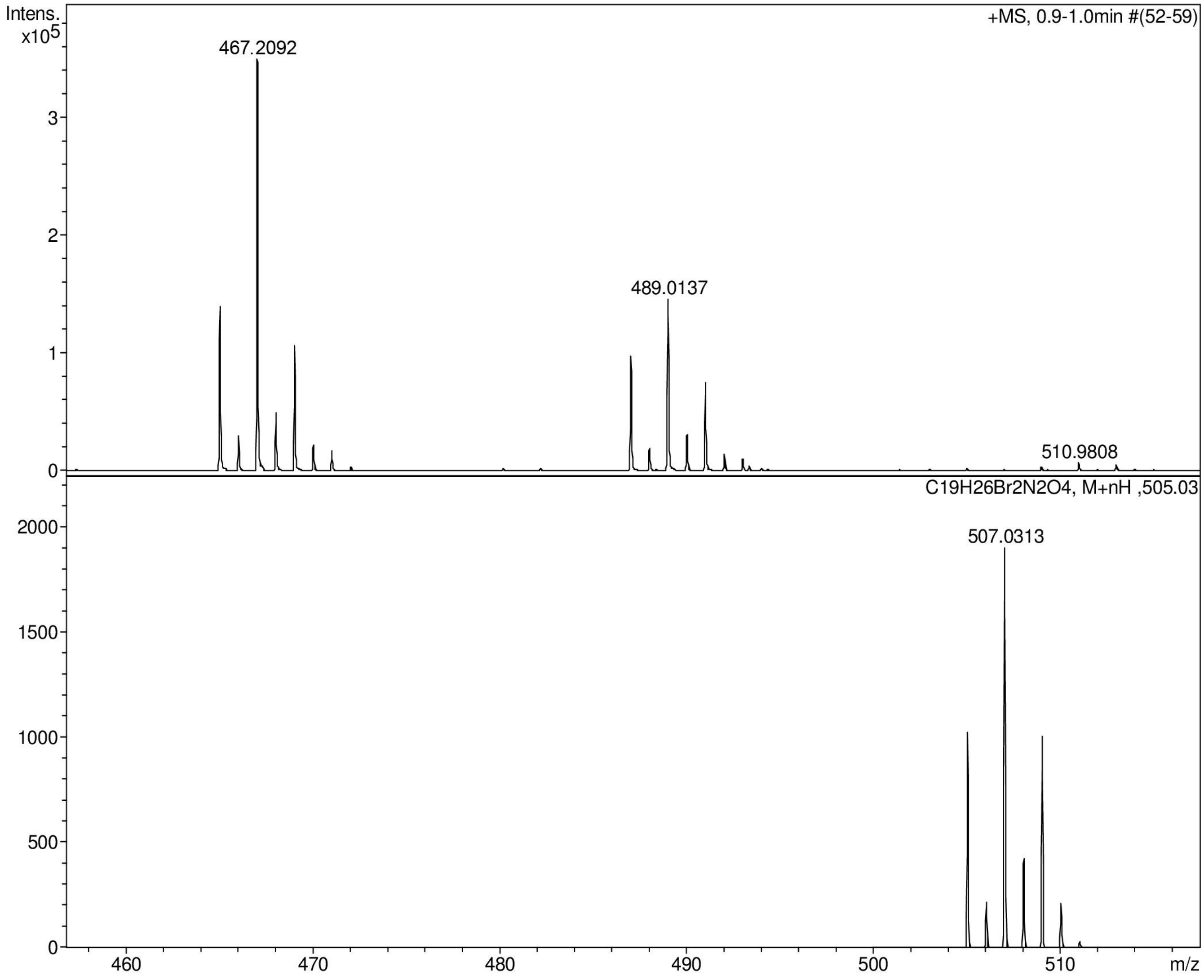

Supplement: Supplementary file 1 [file molecules-30-03710-s001.zip › molecules-3842004-Suppl_Figure S1_11092025.pdf]
